# Parathyroid hormone (1–34) retards the lumbar facet joint degeneration and activates Wnt/β-catenin signaling pathway in ovariectomized rats

**DOI:** 10.1186/s13018-024-04817-6

**Published:** 2024-06-14

**Authors:** Yu Gou, Hetong Li, Xun Sun, Desheng Chen, Faming Tian

**Affiliations:** 1 Department of Orthopaedic Surgery, Tianjin Hospital, Tianjin University, Tianjin, China; 2 School of Public Health, North China University of Science and Technology, Tangshan, China; 3 Department of Orthopaedics, Shenzhen Hospital, Southern Medical University, Shenzhen, China

**Keywords:** Joint degeneration, Lumbar facet joint, Parathyroid hormone, Wnt/β-catenin signaling, Cartilage, Subchondral bone, Ovariectomized rat model

## Abstract

**Purpose:**

Facet joint degeneration (FJD) is a major cause of low back pain. Parathyroid hormone (PTH) (1–34) is commonly used to treat osteoporosis. However, little is known about its effects on FJD induced by estrogen deficiency. This study aims to investigate the effects of PTH (1–34) on FJD induced by estrogen deficiency and the underlying pathogenesis of the disease.

**Methods:**

Forty 3-month-old female Sprague-Dawley rats were randomly divided into four groups: 30 received bilateral ovariectomy (OVX) followed by 12 weeks of treatment with normal saline, PTH (1–34) or 17β-estradiol (E2), and 10 received sham surgery followed by administration of normal saline. Status and Wnt/β-catenin signaling activity in the cartilage and subchondral bone of the L4–L5 FJs and serum biomarkers were analyzed.

**Results:**

Administration of PTH (1–34) and E2 ameliorated cartilage lesions, and significantly decreased MMP-13 and caspase-3 levels and chondrocyte apoptosis. PTH (1–34) but not E2 significantly increased cartilage thickness, number of chondrocytes, and the expression of aggrecan. PTH (1–34) significantly improved microarchitecture parameters of subchondral bone, increased the expression of collagen I and osteocalcin, and decreased RANKL/OPG ratio. E2 treatment significantly increased the OPG level and decreased the RANKL/OPG ratio in the subchondral bone of ovariectomized rats, but it did not significantly improve the microarchitecture parameters of subchondral bone. Wnt3a and β-catenin expression was significantly reduced in the articular cartilage and subchondral bone in OVX rats, but PTH (1–34) could increase the expression of these proteins. E2 significantly increased the activity of Wnt/β-catenin pathway only in cartilage, but not in subchondral bone. The restoration of Wnt/β-catenin signaling had an obvious correlation with the improvement of some parameters associated with the FJs status.

**Conclusion:**

Wnt/β-catenin signaling may be a potential therapeutic target for FJD induced by estrogen deficiency. PTH (1–34) is effective in treating this disease with better efficacy than 17β-estradiol, and the efficacy may be attributed to its restoration of Wnt/β-catenin signaling.

**Supplementary Information:**

The online version contains supplementary material available at 10.1186/s13018-024-04817-6.

## Introduction

Low back pain (LBP) is a significant health concern and a leading global cause of years lived with disability [1, 2]. Intervertebral disc degeneration (IDD) and facet joint (FJ) degeneration (FJD) are the major causes of LBP. It is estimated that the prevalence of FJD pain is 16–40% in the low back [3]. Moreover, the severity and prevalence of FJD pain significantly increase with age, placing a heavy burden on health care systems in aging countries [3, 4]. However, FJD is severely understudied compared to IDD.

Estrogen deficiency is considered a risk factor for some degenerative musculoskeletal diseases [5], including FJD. Estrogen deficiency not only induces FJ articular cartilage degeneration but also leads to osteoporotic subchondral bone, which is characterized by osteoporotic subchondral bone accompanied by high remodeling rates, leading to accelerated cartilage degeneration progression [6, 7]. Although an intervention simultaneously targeting cartilage and subchondral bone is a reasonable choice for the treatment of FJD induced by estrogen deficiency, a lack of effective drugs and therapeutic targets is still a problem.

Wnt/β-catenin signaling, namely canonical Wnt signaling, plays an essential role in regulating development, growth, and disease in joint and bone [8]. In bone tissue of osteoporosis (OP), Wnt/β-catenin signaling is repressed or impaired, which reduces bone mass as a result of bone resorption exceeding bone formation [9–12]. Activating Wnt/β-catenin signaling in bone tissue has therapeutic effects in OP [13]. In cartilage, moderate Wnt/β-catenin signaling activity is critical for chondrocyte proliferation and the maintenance of typical cartilage characteristics [14]. Excessive activation or inhibition of Wnt/β-catenin signaling is involved in cartilage and subchondral bone degeneration, leading to the progression of joint degeneration [15–19]. Thus, Wnt/β-catenin signaling seems to be a promising target in the treatment of FJD induced by estrogen deficiency, but Wnt/β-catenin signaling activity in FJD induced by estrogen deficiency, especially in cartilage, remains to be clarified.

Parathyroid hormone (PTH) 1–34 is the only approved anabolic agent in the clinical treatment of OP. PTH also has the potential to treat joint degeneration, protecting both cartilage and subchondral bone [20]. However, its effect on joint degeneration seems controversial, as some studies have reported that treatment with PTH failed to prevent cartilage deterioration, thickening of the subchondral bone plate, or osteophyte formation [21]. Joint degeneration is a heterogeneous disease, and we assume that the effect of PTH on joint degeneration may depend on its types. Different types of joint degeneration have different pathogenesis, and the corresponding treatment should also vary [22]. Compared with the joint degeneration characterized by subchondral bone sclerosis, PTH may be more suitable for the treatment of joint degeneration with osteoporotic subchondral bone.

Therefore, the aim of this study was to investigate the activity of Wnt/β-catenin signaling in articular cartilage and subchondral bone of FJs in ovariectomized rats, and to explore the effect of PTH (1–34) on FJD induced by estrogen deficiency and compare it with estrogen treatment.

## Methods

### Animals and dosing regimen

All experimental protocols were approved by the Institutional Animal Care and Use Committee. Forty 3-month-old female Sprague-Dawley rats, with an average weight of 269 ± 17 g, were used in the present study. Of these, thirty underwent bilateral ovariectomy (OVX, *n* = 30) and the remaining received sham surgery (Sham, *n* = 10). OVX animals were randomly assigned to three groups: vehicle treatment (OVX, *n* = 10), PTH (1–34) treatment (OVX + PTH, *n* = 10) and 17β-estradiol treatment (OVX + E2, *n* = 10). Rats in the OVX + PTH group were treated with intermittent subcutaneous injections of PTH (1–34) (Sigma-Aldrich Trading Co.Ltd., Shanghai, China) at a dose of 30 µg/kg [23, 24] three days per week post-surgery. As a positive control group, rats in the OVX + E2 group received E2 (Sigma, St. Louis, MO, USA) injections at a dose of 0.1 mg/kg [25, 26] 5 days per week post-surgery. Saline was administered to the animals in the Sham and OVX groups as a placebo 5 days per week post-surgery. All rats were housed in a temperature-controlled room (21 ± 1 °C) with a normal 12-h light/dark cycle and allowed free access to water and a sterilized diet. All animals were euthanized to collect lumbar spine samples and blood after 12 weeks of treatments (The experimental protocol is shown in supplementary Fig. S1).

### Micro-CT analysis

A micro-CT system (SkyScan 1176, Bruker, Kontich, Belgium) was used to scan the superior articular process of the right L4–L5 FJs with a resolution of 17.93-µm per voxel. The region of interest (ROI) was defined as the subchondral cancellous bone with a thickness of 0.4 mm located below the subchondral bone plate to quantify the microarchitecture (Supplementary Fig. S2). The energy and intensity were 50 KeV and 250 µA, respectively. The following morphometric parameters were acquired: bone mineral density (BMD), bone volume/trabecular volume (BV/TV), trabecular separation (Tb.Sp), trabecular thickness (Tb.Th), trabecular number (Tb.N), and structure model index (SMI).

### Histological evaluation and measurement

After micro-CT scanning, all specimens were fixed in 10% neutral buffered formalin and decalcified in 10% EDTA-2Na at room temperature for 10 weeks. Next, the specimens were embedded in paraffin and cut into 6 μm sections, which were stained with toluidine blue for microscopic observation. Histological changes in the cartilage were evaluated using a modified Mankin grading system [27] (Grade 0: intact surface; Grade 1: surface fissure; Grade 2: surface fissure to mid-zone; Grade 3: surface fissures to deep zone; Grade 4: complete destruction). The cartilage thickness of the superior articular process of the right L4–L5 FJs and the number and density of chondrocytes were quantitatively analyzed using Image-Pro Plus software (Media Cybernetics, Inc., USA). All the above data were obtained by two analysts blinded to the allocation, and the average was the final result used for statistical analysis.

### Immunohistochemical analysis

To further clarify the status of cartilage and subchondral bone and the changes in Wnt/β-catenin signaling, immunohistochemistry was performed. Briefly, paraffin sections were sequentially deparaffinized, rehydrated, and incubated with trypsin at 37 °C for 30 min for antigen retrieval. Next, they were treated with 0.3% H_2_O_2_ for 15 min to inactivate endogenous peroxidases and incubated overnight at 4 °C with the following antibodies: aggrecan (AGG) (Cat. No. GTX54920; Gene Tex Inc. USA), collagen-II (Col-II) (II-II6B3 was deposited to the DSHB by Linsenmayer, T.F.), caspase-3 (Cat. No. PB0183; Boster Co., Ltd., Wuhan, China), metalloproteinase-13 (MMP-13) (Cat. No. GTX55707; Gene Tex Inc. USA), a disintegrin and metalloproteinase with thrombospondin motifs 4 (ADAMTS-4) (Cat. No. ab185722; Abcam Inc., USA), collagen-I (Col-I) (Cat. No. BA0325; Boster Co., Ltd., Wuhan, China), osteocalcin (OCN) (Cat. No. AB10911, Merck KgaA, Darmstadt, Germany), receptor activator of nuclear factor-kB ligand (RANKL) (Cat. No. 0747R; Bioss Inc., Beijing, China), osteoprotegerin (OPG) (Cat. No. ab73400; Abcam Inc., USA), Wnt3a (Cat. No. A13601; Abclonal Biotechnology Co., Ltd., Wuhan, China), Axin-2 (Cat. No. ab32197; Abcam Inc., USA), and β-catenin (Cat. No. ab16051; Abcam Inc., USA) (Supplementary Table S1). The remaining procedures were conducted according to the PV-6000 Polink-1 HRP DAB Detection System (ZSGB-BIO Corp., China) and the ZLI-9018 DAB kit (ZSGB-BIO Corp., China). Finally, the sections were counterstained with hematoxylin.

All sections were semi-quantitatively analyzed using Image-Pro Plus software at 100× magnification. The cartilage or subchondral cancellous bone in the middle part of the superior articular process of right L4–L5 FJs at 100× magnification was defined as the region of interest (ROI). The area of the ROI and the sum of the integrated optical density (IOD) of the target protein in the ROI were measured. The average IOD, given in IOD/mm^2^, was the result of the sum of the IOD divided by the ROI area, and measured by two analysts in a blinded manner, the average of which was defined as the final value for statistical analysis.

### Transferase biotin-dUTP nick end labeling (TUNEL) assay

Chondrocyte apoptosis was evaluated using an apoptosis Detection Kit (Cat. No. S7100, EMD Millipore Corporation, Temecula, USA) following the instructions of the manufacturer. Briefly, paraffin sections were sequentially deparaffinized, pretreated with proteinase K (20 µg/mL), quenched in 3% hydrogen peroxide, incubated with equilibration buffer and then with working strength TdT. Next, an anti-digoxigenin conjugate was applied to these sections, and the ZLI-9018 DAB kit (ZSGB-BIO Corp., China) was used for staining under the microscope. Finally, the sections were counterstained with hematoxylin. The ratio of chondrocytes with positive staining to total chondrocytes for each slide was calculated by two blinded observers using Image-Pro Plus software, and the average of the results was defined as the final value.

### Enzyme-linked immunosorbent assay (ELISA)

Blood samples were centrifuged to obtain serum, which was stored at − 80 °C. The serum samples were thawed to room temperature before the test. According to the instructions provided by the manufacturer, serum concentrations of cross linked C-telopeptide of type I collagen (CTX-I) and cartilage oligomeric matrix protein (COMP) were determined using ELISA kits (CSB-E12776r and CSB-E13833r; Cusabio Biotech Co., Ltd., China). The data were acquired with an iMARK Microplate Absorbance Reader (Bio-Rad Laboratories Inc., USA).

### Statistical analysis

All data are presented as means and 95% confidence intervals. The Kolmogorov-Smirnov test was used to decide if the data fulfilled Gaussian distribution. Gaussian distributed data between groups were compared by one-way analysis of variance (ANOVA), followed by Fisher’s least significant difference (LSD) -test or Dunnett’s T3 test for comparison between any two groups, based on the homogeneity of variance. The Kruskal-Wallis H test was used to analyze histological scores and non-Gaussian distributed data, and the Dunn-Bonferroni post hoc test was performed for pairwise comparisons. Pearson’s correlation test was used to analyze the correlation between different parameters. Two-tailed *P* values < 0.05 were considered statistically significant. All statistical analyses were conducted using SPSS software (SPSS 20.0, SPSS Inc.; Chicago, IL, USA).

## Results

### PTH (1–34) partly delayed cartilage degeneration induced by estrogen deficiency

**Fig. 1:**
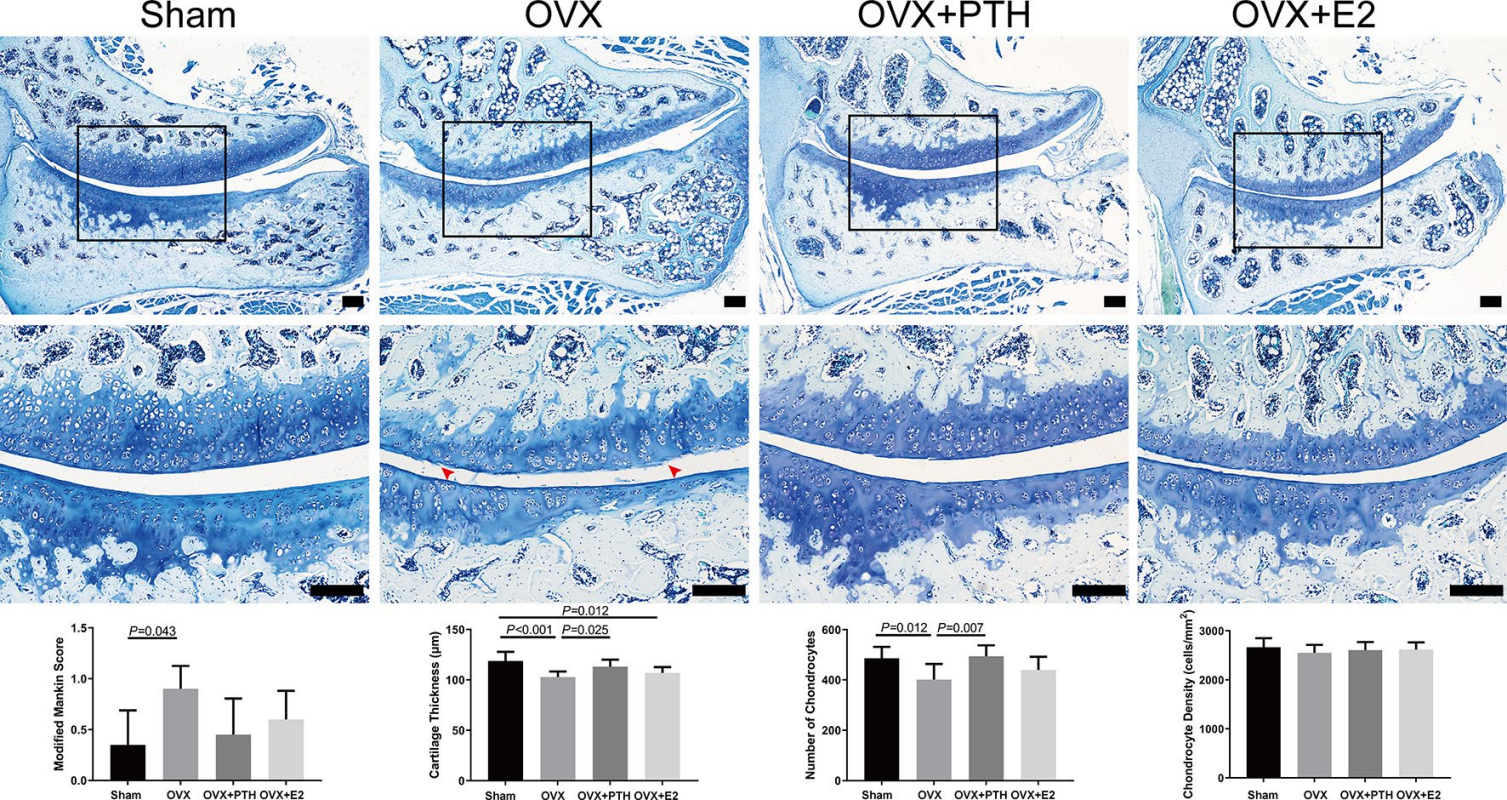
Histological analysis of lumbar L4–L5 FJs (toluidine blue staining). Arrowhead indicates wear and clefts of cartilage surface. Bars = 200 μm. Data are presented as the mean ± 95% CI. The black squares on the upper panels indicate the middle zone of lumbar L4–L5 FJs, which are displayed on the middle panels at 100× magnification. The Kruskal-Wallis H test was used to analyze modified Mankin scores, followed by Dunn-Bonferroni post hoc test to perform pairwise camparation. The ANOVA test was used to analyze the cartilage thickness, number of chondrocytes and chondrocyte density, followed by Fisher’s LSD test to perform pairwise camparation

The articular cartilage in the Sham group rats had a smooth surface, normal cellularity, and abundant chondrocytes, with almost no degeneration (Fig. 1). The OVX group exhibited mild degenerative changes in cartilage characterized by surface irregularities and even superficial clefts, hypocellularity, and thickness reduction (Fig. 1). Administration of PTH (1–34) and E2 partly prevented cartilage degeneration in OVX rats. The OVX group showed a significantly higher modified Mankin score than the Sham group, while there were no significant differences in the modified Mankin scores among the OVX + PTH, OVX + E2, and OVX groups (Fig. 1).

The cartilage thickness and the number of chondrocytes in the OVX group was significantly decreased compared to the Sham and OVX + PTH groups. The cartilage thickness and number of chondrocytes in the OVX + E2 group did not exhibit significant differences compared to those in the OVX + PTH and OVX groups. The chondrocyte density was not significantly different among groups (*P* = 0.737) (Fig. 1).

### PTH (1–34) inhibited cartilage matrix degradation and activated the Wnt/β-catenin pathway

**Fig. 2:**
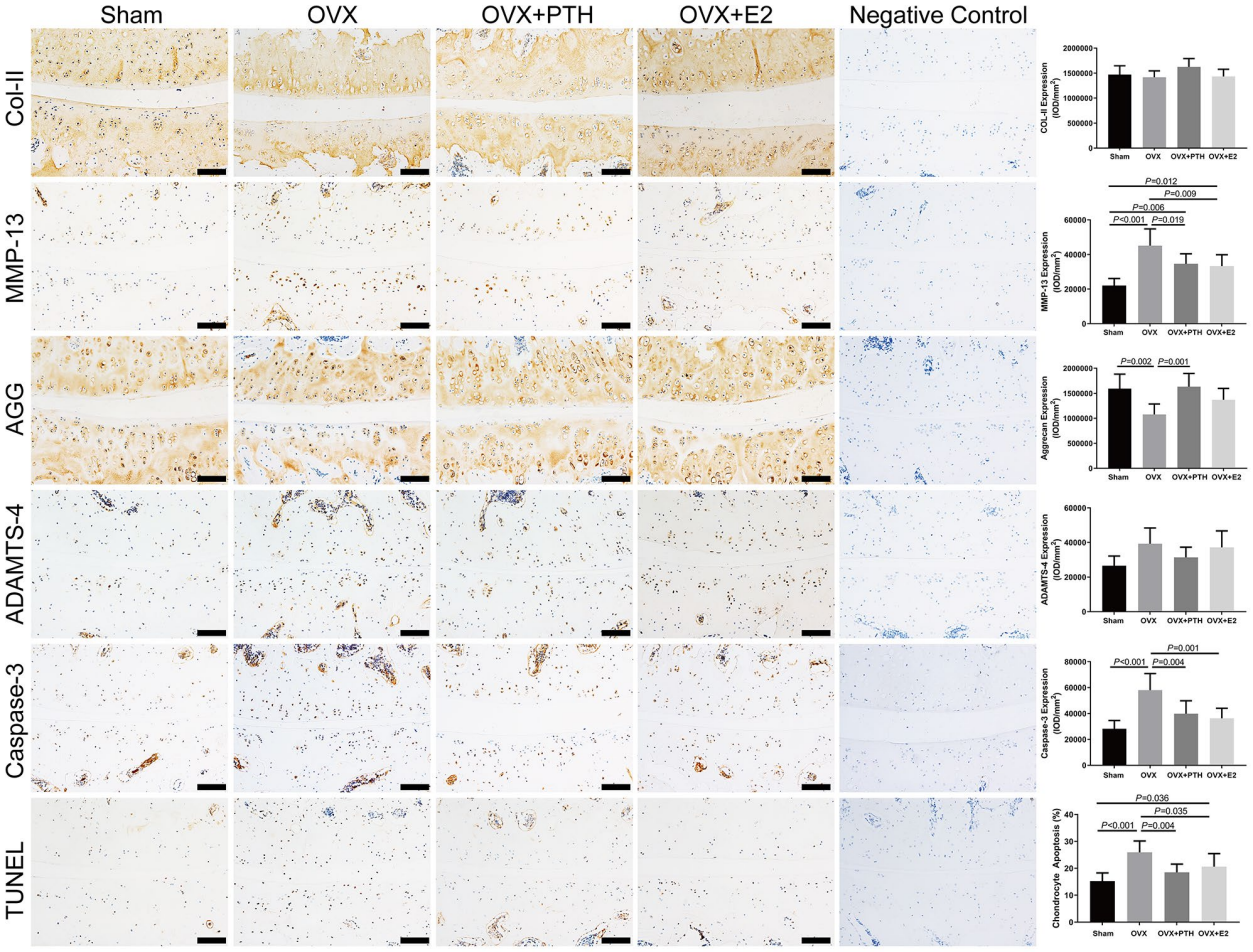
Immunohistochemical staining for caspase-3, AGG, Col-II, MMP-13, and ADAMTS-4, and TUNEL assay in the cartilage of the right L4–5 FJs. Bars = 100 μm. Data are presented as the mean ± 95% CI. The ANOVA test was used to compare multiple means across different groups, followed by Fisher’s LSD test to perform pairwise camparation for caspase-3, AGG, MMP-13, and TUNEL assay

**Fig. 3:**
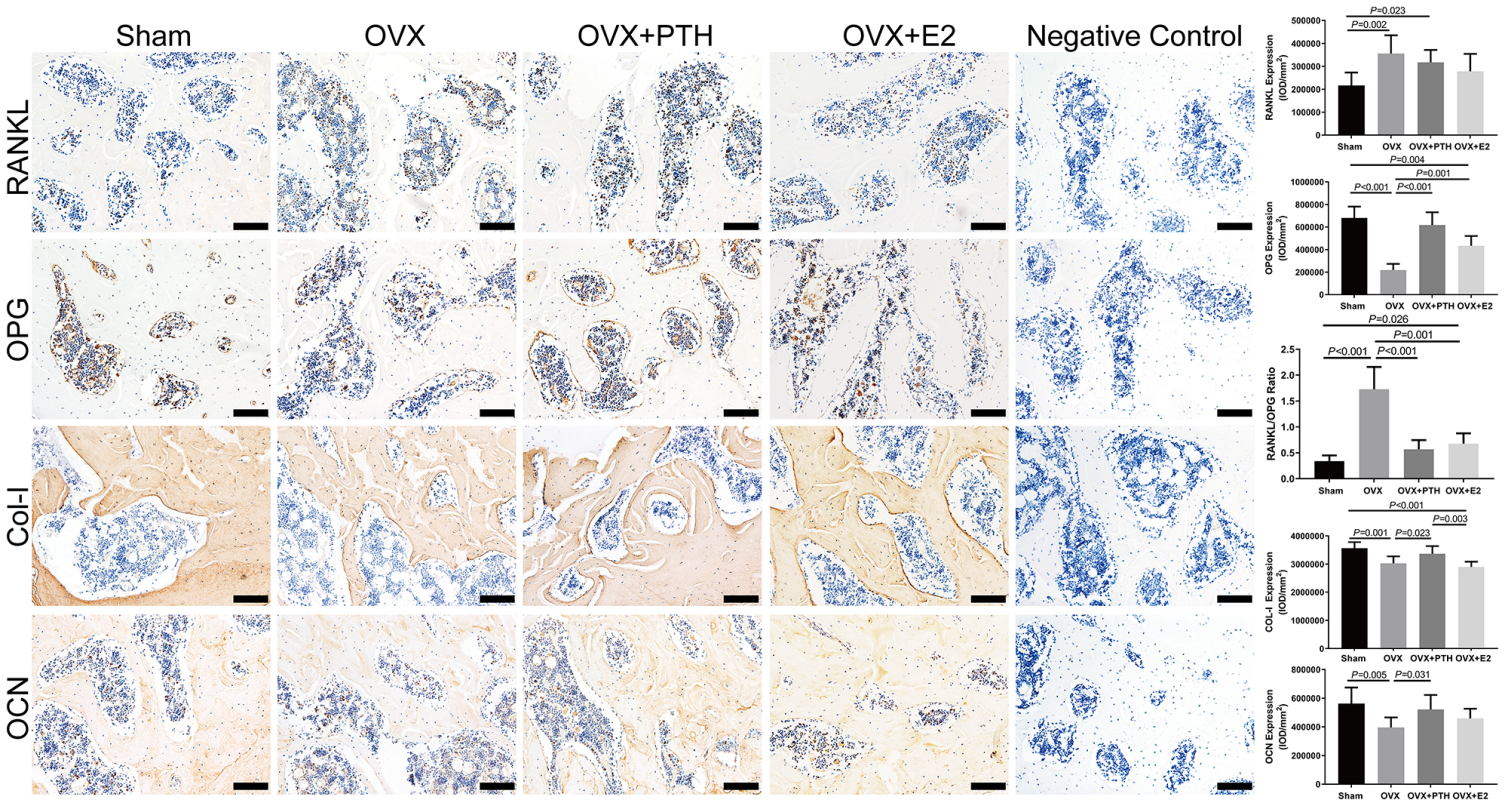
Immunohistochemical staining for RANKL, OPG, Col-I, and OCN in the subchondral bone of the right L4–5 FJs. Bars = 100 μm. Data are presented as the mean ± 95% CI. The ANOVA test was used to compare multiple means across different groups, followed by Fisher’s LSD test to perform pairwise camparation for OCN, RANKL and Col-1, and Dunnett’s T3 test to perform pairwise camparation for OPG and RANKL/OPG ratio

**Fig. 4:**
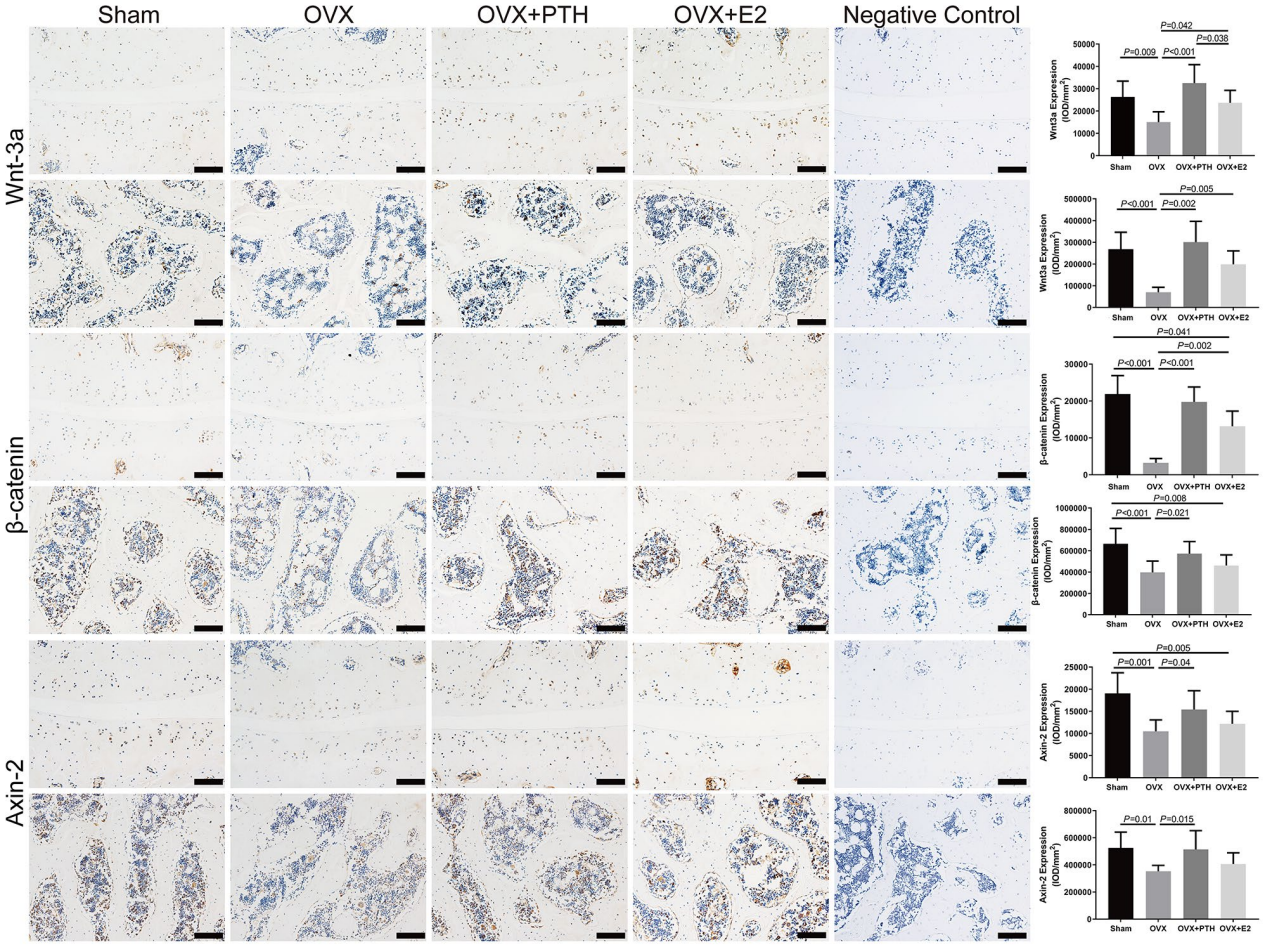
Immunohistochemical staining for Wnt3a, Axin-2 and β-catenin. Representative images were taken from the cartilage or subchondral cancellous bone in the middle part of the superior articular process of right L4–L5 FJs. Bars = 100 μm. Data are presented as the mean ± 95% CI. The ANOVA test was used to compare multiple means across different groups, followed by Fisher’s LSD test to perform pairwise camparation for Axin2 in cartilage and subchondral bone, Wnt3a in cartilage and β-catenin in subchondral bone, and Dunnett’s T3 test to perform pairwise camparation for Wnt3a in subchondral bone and β-catenin in cartilage

In the articular cartilage of FJs, there was no significant difference in Col-II and ADAMTS-4 expression among groups (*P* = 0.15 and = 0.054, respectively) (Fig. 2). AGG was expressed at significantly lower levels in the OVX group than in the Sham and OVX + PTH groups. The expression of AGG in OVX + E2 group was not significantly different from that in the other three groups. Significantly higher MMP-13 levels were observed in the OVX group compared to the Sham, OVX + PTH, and OVX + E2 groups, and in the OVX + PTH and OVX + E2 groups compared to the Sham group (Fig. 2). The OVX group showed significant increases in caspase-3 levels compared to the Sham, OVX + PTH, and OVX + E2 groups (Fig. 2). Wnt3a was expressed at significantly lower levels in the OVX group than in the Sham, OVX + PTH, and OVX + E2 groups, and in the OVX + E2 group than in the OVX + PTH group (Fig. 3). β-catenin expression levels in the cartilage tissue were significantly lower in the OVX group than in the Sham, OVX + PTH and OVX + E2 groups. Axin-2 was expressed at significantly lower levels in the OVX group than in the Sham and OVX + PTH groups, and in the OVX + E2 group than in the Sham group (Fig. 4).

In subchondral bone, RANKL was expressed at significantly higher levels in the OVX and OVX + PTH groups than in the Sham group, but no significant differences were observed among the OVX, OVX + E2 and OVX + PTH groups (Fig. 3). The OVX group showed significant decreases in OPG levels but increases in RANKL/OPG ratio compared to the Sham, OVX + PTH, and OVX + E2 groups. The OVX group showed significantly lower expression levels of Col-I and OCN than the Sham and OVX + PTH groups, and the OVX + PTH showed significantly higher expression levels of Col-I than the OVX + E2 group (Fig. 3). Significantly lower Wnt3a, β-catenin and Axin-2 levels were observed in the OVX group than those in the Sham and OVX + PTH groups (Fig. 4). The expression levels of Wnt3a in the OVX + E2 group were significantly higher than those in the OVX group, while the expression level of β-catenin in the OVX + E2 group was significantly lower than that in the Sham group (Fig. 4).

### PTH (1–34) inhibited chondrocyte apoptosis

Chondrocyte apoptosis was evaluated by TUNEL staining on sections from each group. The TUNEL-positive chondrocytes showed brown staining and were easily identified. The percentage of TUNEL-positive chondrocytes was significantly higher in the OVX group than that in the Sham, OVX + PTH, and OVX + E2 groups, and in the OVX + E2 group than in the Sham group (Fig. 2).

### PTH (1–34) improved the microarchitecture of subchondral bone

**Fig. 5:**
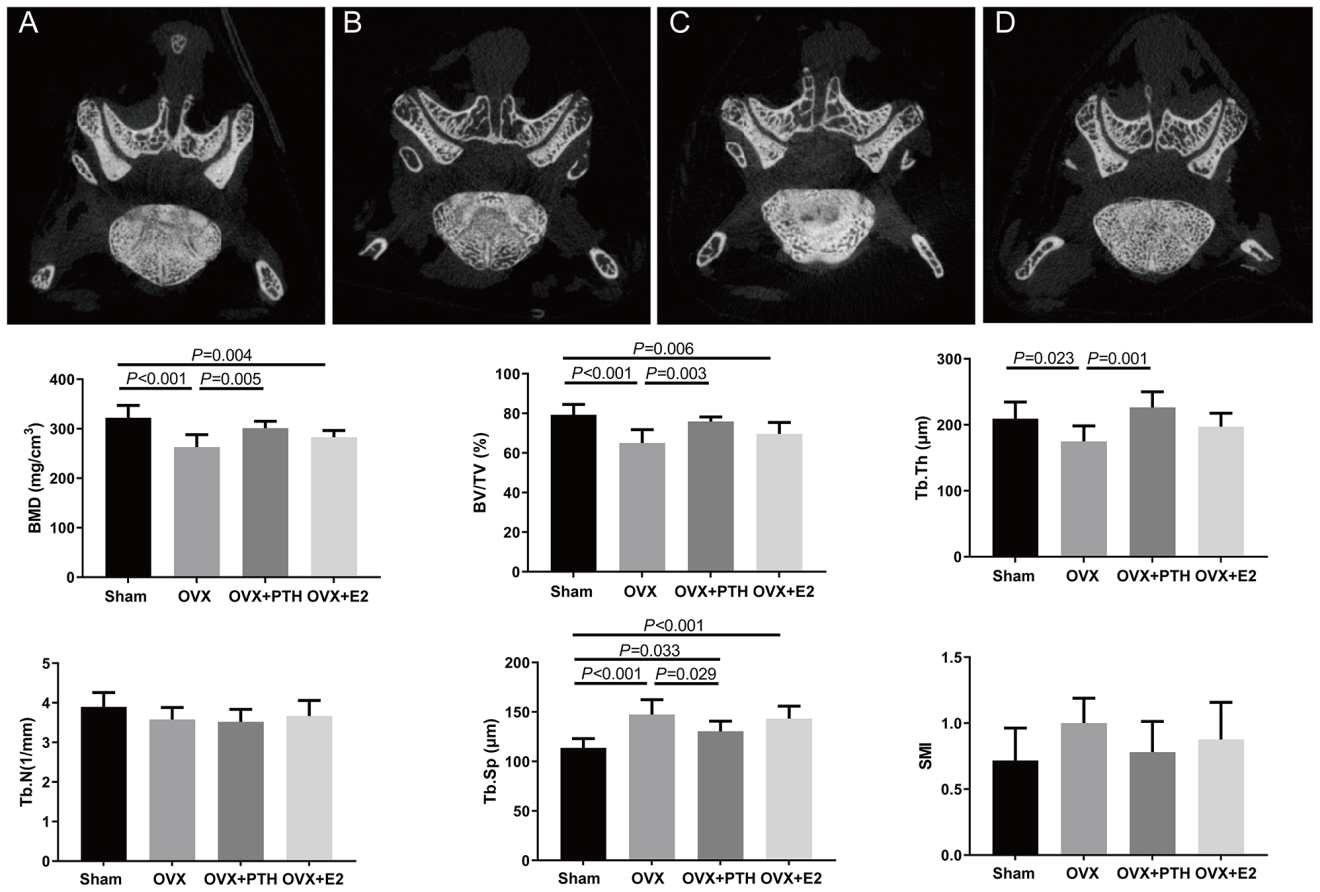
2-Dimension reconstruction of the right L4–5 FJs and morphological parameters of subchondral bone. (**A**) Sham group, (**B**) OVX group, (**C**) OVX + PTH group, and (**D**) OVX + E2 group. Data are presented as the mean ± 95% CI. The ANOVA test was used to compare multiple means across different groups, followed by Fisher’s LSD test to perform pairwise camparation for BV/TV, Tb.Sp and Tb.Th, and Dunnett’s T3 test to perform pairwise camparation for BMD

The OVX group displayed significantly lower BMD, BV/TV, and Tb.Th values than those in the Sham and OVX + PTH groups, whereas the Tb.Sp value was significantly higher in the OVX group than in the Sham and OVX + PTH groups (Fig. 5). The BMD and BV/TV values in the OVX + E2 group were significantly lower than those in the Sham group, while the Tb.Sp value was significantly higher in the OVX + E2 group than in the Sham group. Besides, the Tb.Sp value were significantly increased in the OVX + PTH group compared to those in the sham group. There was no significant difference in Tb.N and SMI among groups (*P* = 0.333 and = 0.273, respectively) (Fig. 5).

### PTH (1–34) did not change significantly decrease the serum COMP and CTX-I levels

**Fig. 6:**
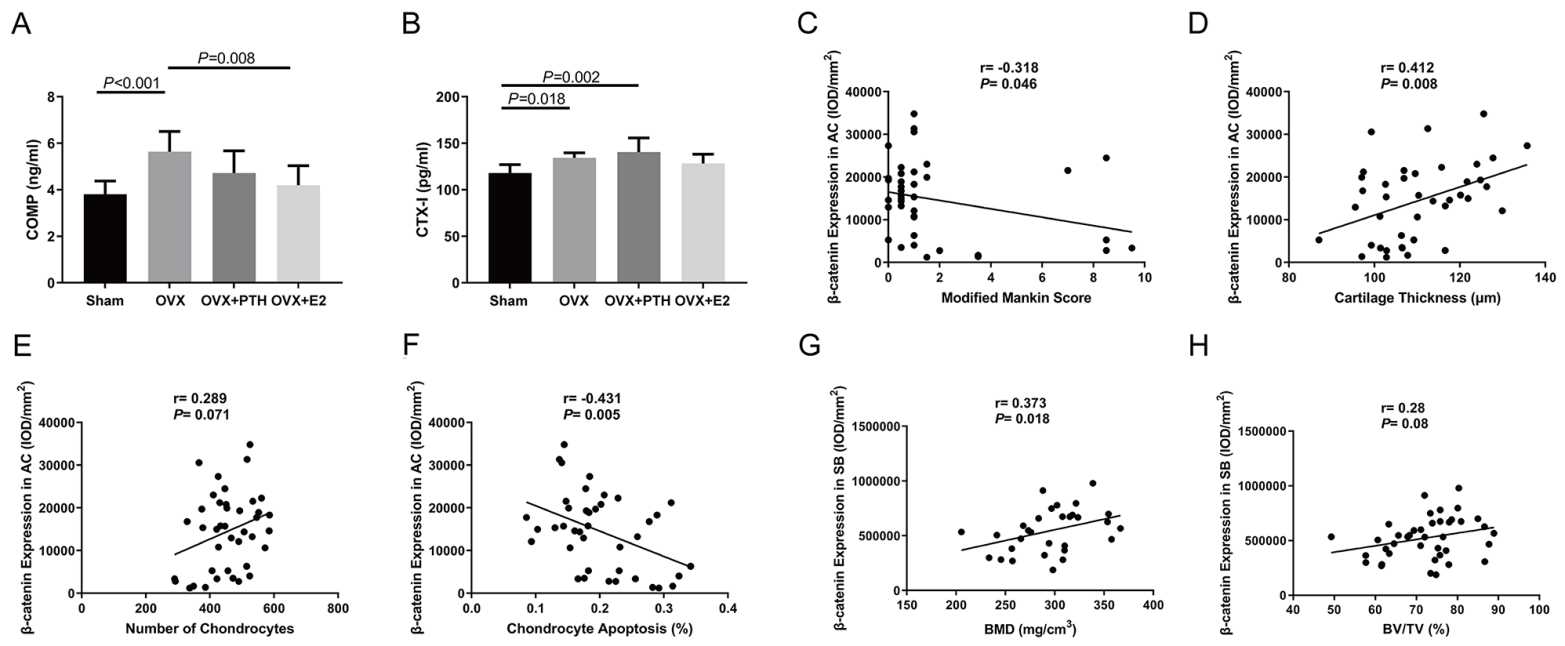
Serum biomarker concentrations and correlation analysis. (**A**-**B**) Serum concentrations of COMP and CTX-I in each group. (**C**-**F**) Analysis of correlations between β-catenin expression in articular cartilage and modified Mankin score, cartilage thickness, number of chondrocytes, and chondrocyte apoptosis, and (**G** and **H**) between β-catenin expression in subchondral bone and BMD and BV/TV values (Pearson´s coefficient (r) and two-tailed *P*-value). AC, articular cartilage; SB, subchondral bone. Data are presented as the mean ± 95% CI. The ANOVA test was used to analyze the serum concentrations of COMP and CTX-I, followed by Fisher’s LSD test to perform pairwise camparation. Pearson’s correlation test was used to analyze the correlation between different parameters

The serum COMP level in the OVX group was significantly higher than that in the Sham and OVX + E2 groups, but showed no significant difference compared to the OVX + PTH group (*P* = 0.083) (Fig. 6A). The serum CTX-I level in the Sham group was significantly lower than that in the OVX and OVX + PTH groups (Fig. 6B).

### Wnt/β-catenin pathway was strongly associated with the health of cartilage and subchondral bone

The level of β-catenin in cartilage had a positive correlation with cartilage thickness and the number of chondrocytes (Fig. 6D and E), but a significant negative correlation with the modified Mankin score and the percentage of TUNEL-positive cells (Fig. 6C and F). The level of β-catenin in subchondral bone had a significant positive correlation with BMD (Fig. 6G), but it had no significant correlation with BV/TV (Fig. 6H).

## Discussion

To the best of our knowledge, this is the first study to demonstrate the inhibition of Wnt/β-catenin signaling activity not only in subchondral bone but also in the articular cartilage of degenerative FJs in OVX rats. Moreover, PTH (1–34) showed protective effects on both cartilage and subchondral bone of degenerative FJs in OVX rats, which may be related to its activation of the Wnt/β-catenin signaling pathway.

Joint degeneration is now considered a disease affecting the whole joint involving changes in the articular cartilage, subchondral bone, capsule, and synovial membrane, ultimately resulting in joint failure [28, 29]. Although previous studies have been conducted on FJD induced by estrogen deficiency [7, 30], the mechanism of the disease remains poorly understood. In the present study, the articular cartilage of FJs in the Sham group had a smooth surface with almost no degenerative changes. The OVX group exhibited mild cartilage damage characterized by surface irregularities and even superficial clefts, accompanied with a significantly higher modified Mankin score. Cartilage thickness and AGG levels in the extracellular matrix (ECM) were significantly decreased, but the expression of matrix-degrading enzymes (MMP-13) was significantly higher in the OVX group than that in the Sham group. These results indicated that the imbalance of cartilage ECM metabolism is an important cause of cartilage degeneration in FJD induced by estrogen deficiency. We found a significantly decreased number of chondrocytes, a significantly increased percentage of TUNEL-positive cells, and increased expression of caspase-3 in the OVX group compared with the Sham group. Chondrocytes are the sole cell type in articular cartilage and play an irreplaceable role in maintaining cartilage health. We, therefore, believe that the decreased chondrocyte number caused by excessive cell apoptosis is one of the main factors leading to cartilage degeneration in FJs of OVX rats, which may further trigger or exacerbate the imbalance of cartilage ECM metabolism.

The most surprising finding in our study was that Wnt3a and β-catenin were expressed at significantly lower levels in the articular cartilage of FJs in the OVX group than in the Sham group, indicating significant inhibition of Wnt/β-catenin signaling. Ours is the first study to investigate the activity of Wnt/β-catenin signaling in the articular cartilage of FJD induced by estrogen deficiency. Excessive activation and inhibition of Wnt/β-catenin signaling are involved in the degeneration of cartilage and subchondral bone. There seem to be differences in the Wnt/β-catenin signaling activity in articular cartilage among different types of joint degeneration. Wnt/β-catenin signaling was excessively activated in the articular cartilage of joint degeneration induced by trauma [15, 16] or collagenase [17]. However, it was largely inhibited in the joint degeneration induced by compressive mechanical force, accompanied by significantly decreased cartilage thickness, chondrocyte number, and chondrocyte proliferation markers, and significantly increased chondrocyte apoptosis [18]. An in vitro study found impaired growth and increased apoptosis of chondrocytes by suppressing nuclear β-catenin accumulation and Akt phosphorylation [31]. Moreover, inhibiting Wnt/β-catenin signaling in articular chondrocytes caused increased cell apoptosis and articular cartilage destruction in Col2a1-ICAT-transgenic mice [19]. Therefore, we considered that inhibited Wnt/β-catenin signaling plays an important role in promoting or even initiating cartilage degeneration of FJs in OVX rats by increasing chondrocyte apoptosis. Correlation analysis results showed that the β-catenin level in cartilage significantly correlated with several indicators related to chondrocyte apoptosis and cartilage degeneration, which partly substantiates our view.

This study demonstrates that treatment with PTH (1–34) protected the structure of the articular cartilage of FJs in OVX rats, represented by decreased histological score and increased cartilage thickness and chondrocyte number. This finding may be attributed to the restoration of ECM metabolism by increasing AGG expression and decreasing matrix-degrading enzyme expression. PTH (1–34) also showed protective effects on chondrocytes. Chang et al. indicated that PTH (1–34) treatment reduces dexamethasone-induced terminal differentiation and apoptosis of articular chondrocytes [32]. Chang et al. found that PTH (1–34) inhibits the terminal differentiation of human articular chondrocytes in vitro and inhibits the progression of osteoarthritis in rats in vivo [33]. This finding concurs well with our study, which showed that PTH (1–34) preserves chondrocytes by inhibiting cell apoptosis and increasing their number. Hormone therapy seems to be a promising approach for treating diseases caused by estrogen deficiency, but its clinical application is limited given its uncertainty on women’s health (such as increased risk of thrombosis, asthma, meningioma, thyroid, breast, and ovarian cancer) [34, 35]. Previous findings have shown that estrogen can protect cartilage and inhibit intervertebral disc degeneration by suppressing catabolic activity of protease (MMP-13) [36, 37]. Consistent with previous studies [36–38], our study found that estrogen improved histological score, and inhibited MMP-13 expression and chondrocyte apoptosis. However, compared with PTH 1–34, estrogen treatment did not significantly increase cartilage thickness and chondrocyte number, potentially attributable to its limited capability to elevate AGG expression levels and consequently stimulate cartilage matrix synthesis.

Wnt3a and β-catenin expression in the FJs cartilage in the OVX + PTH and OVX + E2 groups was significantly higher than that in the OVX group. The ability of PTH and estrogen to activate the Wnt/β-catenin pathway has been proved in previous studies [39, 40]. Jia et al. found that PTH and estrogen can enhance Wnt/β-catenin pathway activity in nucleus pulposus and retard disc degeneration in OVX rats [39]. Tian et al. indicated that PTH induced osteoblast differentiation mainly through the activation of the Wnt/β-catenin pathway in osteoblastic MC3T3-E1 cells [40]. To the best of our knowledge, ours is the first study to show that PTH and estrogen can enhance the activity of Wnt/β-catenin signaling in chondrocytes of OVX animals. Suppressing the Wnt/β-catenin pathway promoted chondrocyte apoptosis and cartilage matrix degradation, but the restoration of the Wnt/β-catenin pathway largely recovered chondrocyte proliferation and prevented cartilage deterioration [18, 31]. Therefore, we believe that activating Wnt/β-catenin signaling in chondrocytes is one of the potential mechanisms for PTH (1–34) to exert its protective effects on the cartilage in FJD induced by estrogen deficiency.

In the present study, the rats in the OVX group had significantly decreased BMD, BV/TV, and Tb.Th values, significantly lower levels of OPG, Col-I, and OCN, but significantly increased Tb.Sp value and RANKL and RANKL/OPG ratio compared with the sham-operated rats. These results demonstrated that the subchondral bone was undergoing abnormal bone remodeling, with bone loss exceeding bone growth. Inhibited Wnt/β-catenin signaling activity was found in the subchondral bone of FJs in OVX rats, which was consistent with that seen in the subchondral bone of degenerative knee joints in OVX rats [41]. However, in other types of joint degeneration, such as joint degeneration induced by trauma [42, 43], Wnt/β-catenin signaling was over-activated in the subchondral bone, causing subchondral bone sclerosis. Usually, Wnt/β-catenin signaling promotes bone formation by directing mesenchymal progenitor cell differentiation along an osteoblast lineage, stimulating osteoblast differentiation and proliferation, and prolonging the survival of osteoblasts and uncommitted osteoblast progenitors [44, 45]. Wnt/β-catenin signaling also inhibits osteoclast differentiation by regulating osteoblast expression of OPG and RANKL/OPG ratio [46, 47]. Consequently, drugs activating Wnt/β-catenin signaling in bone tissues are being regarded as promising options for the treatment of osteoporotic changes [13].

Although PTH stimulates bone formation and bone resorption, the net effect on bone mass depends on the duration and periodicity of exposure [48]. Intermittent treatment with PTH (1–34) markedly improved the microstructure of subchondral bone in OVX rats, significantly increasing BMD, BV/TV, and Tb.Th values, but decreasing Tb.Sp value in our study. We also found that Wnt3a and β-catenin expression significantly increased in the subchondral bone in the OVX + PTH group. While the mechanism of PTH anabolic function is complex, it is partly related to the activation of Wnt/β-catenin signaling [49]. PTH administration has been found to reduce the mRNA and protein levels of Wnt antagonists SOST/sclerostin and Dickkopf-1 and concurrently increase bone mass [50, 51]. PTH-induced osteoblast differentiation occurs mainly through activation of the Wnt/β-catenin pathway [40]. The activation of Wnt/β-catenin signaling allows the accumulated β-catenin to bind T-cell-specific transcription factor and lymphoid enhancing factor, leading to the transcription of Wnt-specific osteoblast differentiation genes [52]. PTH (1–34) also significantly increased the expression of Col-I and OCN, but significantly decreased the RANKL/OPG ratio in the subchondral bone of OVX rats. Col-I and OCN are secreted by osteoblasts and play a crucial role in bone formation. The ratio of RANKL/OPG is an important factor in determining bone mass and integrity. Thus, these results indicated that PTH (1–34) has protective effects on the subchondral bone of FJs in OVX rats.

Our study found that the protective effects of estrogen on subchondral bone were weaker than that of PTH (1–34). Although estrogen significantly decreased the RANKL/OPG ratio and increased the expression level of OPG in the subchondral bone of the ovariectomized rats, it did not significantly improve the morphological parameters of subchondral bone. Previous studies have shown that estrogen has protective effects on subchondral bone, but some studies suggest that it may damage the health of subchondral bone [38, 53]. Therefore, the effect of estrogen on subchondral bone needs to be further studied. In addition, estrogen did not significantly increase the expression level of β-catenin (the key nuclear effector of Wnt/β-catenin signaling in the nucleus) in subchondral bone, which may also be one of the reasons why it does not provide as much benefit to subchondral bone as PTH (1–34).

One limitation of the present study is that the body weight, food intake and uterine weight were not recorded and assessed for all animals, which could provide more information on the effects of OVX, PTH (1–34) and E2 on this animal model.

In summary, the results of the present study suggest that PTH (1–34) is an effective drug for the treatment of FJD induced by estrogen deficiency. PTH (1–34) exerts protective effects on the cartilage and subchondral bone of lumbar FJs in OVX rats and is more effective than 17β-estradiol, which may be attributed to the restoration of suppressed Wnt/β-catenin signaling in these structures. Moreover, we found for the first time that Wnt/β-catenin signaling activity was inhibited in the cartilage and subchondral bone of degenerative FJs in OVX rats. Furthermore, the restoration of Wnt3a and β-catenin expression has an obvious correlation with the improvement of some important parameters associated with the status of cartilage and subchondral bone. These findings indicate that Wnt/β-catenin signaling is a potential therapeutic target for FJD induced by estrogen deficiency and can aid in investigating the pathogenesis and pharmaceutical therapies for this disease. Future studies are needed to investigate the effects of other osteoporosis drugs, such as romosozumab, on articular cartilage and Wnt/β-catenin signaling in osteoporosis patients.

### Electronic supplementary material

Below is the link to the electronic supplementary material.

Supplementary Material 1

Supplementary Material 2

Supplementary Material 3

## Data Availability

All data generated or analyzed during this study are included in this published article and its supplementary information files.
