# Supplementary material for: Parathyroid hormone (1–34) retards the lumbar facet joint degeneration and activates Wnt/β-catenin signaling pathway in ovariectomized rats
Source: J Orthop Surg Res. 2024 Jun 14;19:352. doi: 10.1186/s13018-024-04817-6 (PMC11177467; doi:10.1186/s13018-024-04817-6)
Supplement: Supplementary file 1 — Supplementary Material 1 [file 13018_2024_4817_MOESM1_ESM.docx]

**Table S1**. The antibodies used in this study are listed according to the Antibody Registry.

| **Antibody RRID** | **Antibody name** | **Target Antigen** | **Vendor** | **Vendor identifier** | **Host organism** | **Clonality** | **Dilution** | **Evaluation method** |
| --- | --- | --- | --- | --- | --- | --- | --- | --- |
| AB_2887906 | Aggrecan antibody | human, mouse, rat | Gene Tex | GTX54920 | Rabbit | Polyclonal antibody | 1:500 | loss or retained staining quantified by Image pro plus software |
| AB_528165 | Mouse Anti-Chicken collagen type II Monoclonal Antibody | Collagen type II epitope: Full length protein; Epitope lies within the triple-helical domain of chick collagen type II. avian, broad species, chicken, fish, goat, human, mouse, ovine, shark, xenopus, zebrafish | DSHB | II-II6B3 | Mouse | Monoclonal Antibody | 1:100 | same as above |
| AB_2890203 | Anti-CASP3(P17) Antibody | Caspase3 human, mouse, rat | Boster | PB0183 | Rabbit | Polyclonal antibody | 1:200 | same as above |
| AB_2891223 | metalloproteinase-13 (MMP-13) antibody | MMP-13 human, mouse | Gene Tex | GTX55707 | Rabbit | Polyclonal antibody | 1:200 | same as above |
| AB_2891225 | Anti-ADAMTS4 antibody | ADAMTS4 mouse, rat, human | Abcam | ab185722 | Rabbit | Polyclonal antibody | 1:200 | same as above |
| AB_2891224 | Anti-COL1A1 Antibody | COL1A1 human, mouse, rat | Boster | BA0325 | Rabbit | Polyclonal antibody | 1:100 | same as above |
| AB_1587337 | Anti-Osteocalcin antibody | Osteocalcin human, mouse, rat | Millipore | AB10911 | Rabbit | Polyclonal antibody | 1:500 | same as above |
| AB_11118767 | Rabbit Anti-OPGL/RANKL/ODF Polyclonal Antibody, HRP Conjugated | Rabbit OPGL/RANKL/ODF HRP human, mouse, canine, rat, human, mouse, rat, dog | Bioss | bs-0747R | Rabbit | Polyclonal antibody | 1:200 | same as above |
| AB_1640751 | Osteoprotegerin antibody | Osteoprotegerin human, predicted to work with mouse, rat, sheep, cow, pig | Abcam | ab73400 | Rabbit | Polyclonal antibody | 1:500 | same as above |
| AB_2760464 | WNT3A Polyclonal Antibody | WNT3A Human, Rat | Abclonal | A13601 | Rabbit | Polyclonal antibody | 1:100 | same as above |
| AB_443301 | beta Catenin antibody | beta Catenin antibody rat, mouse, xenopus/amphibian, human, mouse, rat, xenopus laevis | Abcam | ab16051 | Rabbit | Polyclonal antibody | 1:300 | same as above |
| AB_2290204 | Axin 2 antibody | Axin 2 antibody human, mouse, rat, | Abcam | ab32197 | Rabbit | Polyclonal antibody | 1:200 | same as above |
