# Supplementary material for: Parathyroid hormone (1–34) retards the lumbar facet joint degeneration and activates Wnt/β-catenin signaling pathway in ovariectomized rats
Source: J Orthop Surg Res. 2024 Jun 14;19:352. doi: 10.1186/s13018-024-04817-6 (PMC11177467; doi:10.1186/s13018-024-04817-6)
Supplement: Supplementary file 2 — Supplementary Material 2 [file 13018_2024_4817_MOESM2_ESM.pptx]

## Slide 1
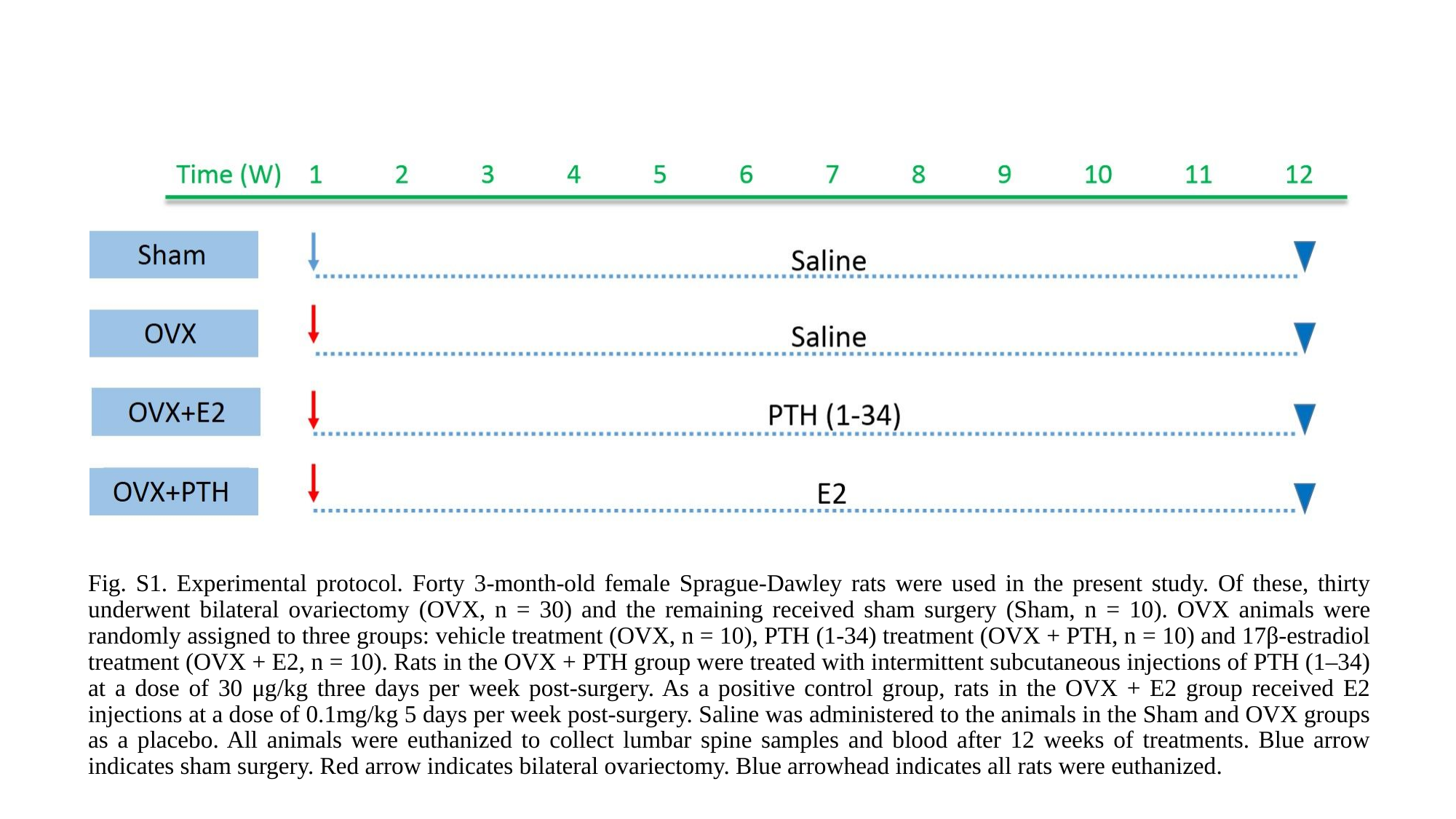

# Fig. S1. Experimental protocol. Forty 3-month-old female Sprague-Dawley rats were used in the present study. Of these, thirty underwent bilateral ovariectomy (OVX, n = 30) and the remaining received sham surgery (Sham, n = 10). OVX animals were randomly assigned to three groups: vehicle treatment (OVX, n = 10), PTH (1-34) treatment (OVX + PTH, n = 10) and 17β-estradiol treatment (OVX + E2, n = 10). Rats in the OVX + PTH group were treated with intermittent subcutaneous injections of PTH (1–34) at a dose of 30 μg/kg three days per week post-surgery. As a positive control group, rats in the OVX + E2 group received E2 injections at a dose of 0.1mg/kg 5 days per week post-surgery. Saline was administered to the animals in the Sham and OVX groups as a placebo. All animals were euthanized to collect lumbar spine samples and blood after 12 weeks of treatments. Blue arrow indicates sham surgery. Red arrow indicates bilateral ovariectomy. Blue arrowhead indicates all rats were euthanized.
