# Supplementary material for: Parathyroid hormone (1–34) retards the lumbar facet joint degeneration and activates Wnt/β-catenin signaling pathway in ovariectomized rats
Source: J Orthop Surg Res. 2024 Jun 14;19:352. doi: 10.1186/s13018-024-04817-6 (PMC11177467; doi:10.1186/s13018-024-04817-6)
Supplement: Supplementary file 3 — Supplementary Material 3 [file 13018_2024_4817_MOESM3_ESM.pptx]

## Slide 1
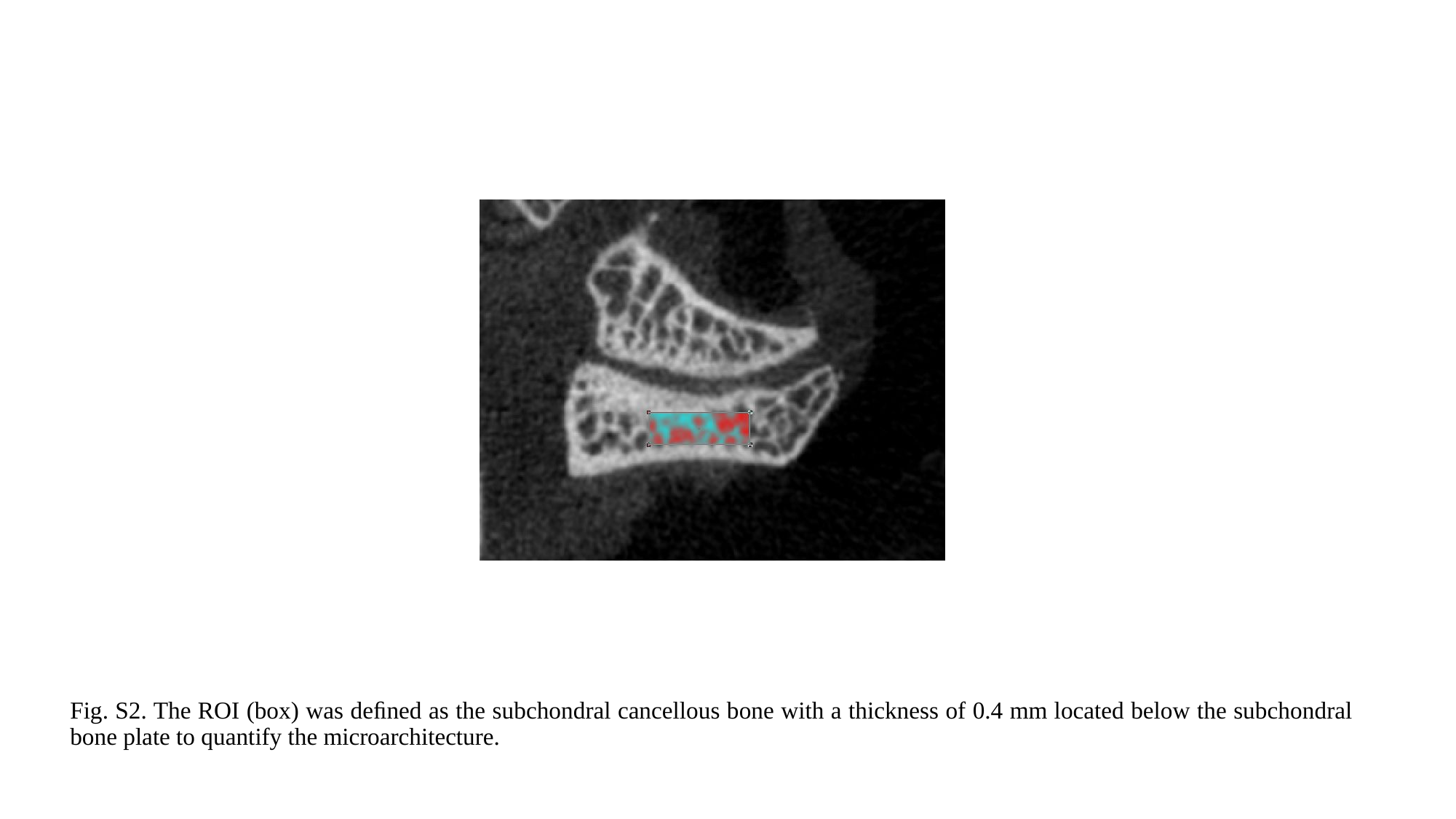

# Fig. S2. The ROI (box) was deﬁned as the subchondral cancellous bone with a thickness of 0.4 mm located below the subchondral bone plate to quantify the microarchitecture.
